# Biostimulant Action of Dissolved Humic Substances From a Conventionally and an Organically Managed Soil on Nitrate Acquisition in Maize Plants

**DOI:** 10.3389/fpls.2019.01652

**Published:** 2020-01-15

**Authors:** Tihana Vujinović, Laura Zanin, Silvia Venuti, Marco Contin, Paolo Ceccon, Nicola Tomasi, Roberto Pinton, Stefano Cesco, Maria De Nobili

**Affiliations:** ^1^Dipartimento di Scienze Agroalimentari, Ambientali e Animali, University of Udine, Udine, Italy; ^2^Faculty of Science and Technology, Free University of Bolzano, Bolzano, Italy

**Keywords:** dissolved organic matter, nitrate uptake, organic farming, root gene expression, soil organic matter

## Abstract

Conversion of conventional farming (CF) to organic farming (OF) is claimed to allow a sustainable management of soil resources, but information on changes induced on dissolved organic matter (DOM) are scarce. Among DOM components, dissolved humic substances (DHS) were shown to possess stimulatory effects on plant growth. DHS were isolated from CF and OF soil leacheates collected from soil monolith columns: first in November (bare soils) and then in April and June (bare and planted soils). DHS caused an enhancement of nitrate uptake rates in maize roots and modulated several genes involved in nitrogen acquisition. The DHS from OF soil exerted a stronger biostimulant action on the nitrate uptake system, but the first assimilatory step of nitrate was mainly activated by DHS derived from CF soil. To validate the physiological response of plants to DHS exposure, real-time RT-PCR analyses were performed on those genes most involved in nitrate acquisition, such as *ZmNRT2.1*, *ZmNRT2.2*, *ZmMHA2* (coding for two high-affinity nitrate transporters and a PM H^+^-proton pump), *ZmNADH:NR*, *ZmNADPH:NR*, and *ZmNiR* (coding for nitrate reductases and nitrite reductase). All tested DHS fractions induced the upregulation of nitrate reductase (NR), and in particular the OF2 DHS stimulated the expression of both tested transcripts encoding for two NR isoforms. Characteristics of DHS varied during the experiment in both OF and CF soils: a decrease of high molecular weight fractions in the OF soil, a general increase in the carboxylic groups content, as well as diverse structural modifications in OF vs. CF soils were observed. These changes were accelerated in planted soils. Similarity of chemical properties of DHS with the more easily obtainable water-soluble humic substance extracted from peat (WEHS) and the correspondence of their biostimulant actions confirm the validity of studies which employ WEHS as an easily available source of DHS to investigate biostimulant actions on agricultural crops.

## Introduction

Organic farming (OF) is claimed to mitigate the impact of agricultural practices on ecosystems while satisfactorily sustaining crop yields; in this framework, the crucial role of soil organic matter (SOM) has been thoroughly investigated (Schrama et al., 2018).

The meta-analysis carried out by Bai et al. (2018) on several long-term experiments confirms that SOM content is larger in soils managed according to OF principles rather than to conventional farming (CF). However, the authors suggested that quantitative differences alone might not provide full reason for the several benefits induced by organic farming on soil resilience and on the sustainability of soil biological fertility.

Conventional farming, on the other hand, often results in reduced biological fertility with a decreased capacity of soils to support healthy crop growth. Reasons for this are still poorly understood: loss of SOM, nutrient imbalance, and massive use of agrochemicals are proven to contribute, but do not fully explain the observed outcomes. Climate change is expected to exacerbate abiotic stresses, so there is a pressing need to better understand the mechanisms of soil–plant–microorganism interactions that support the resilience of not cultivated and organically managed soils and crops (Clair and Lynch, 2010).

Dissolved organic matter (DOM) is defined as the fraction of SOM dissolved in the soil liquid phase, therefore representing the most mobile and bioavailable pool of soil organic matter. DOM includes molecules with diverse degrees of biological recalcitrance, from simple labile plant and microbial metabolites (amino acids and sugars) to more persistent compounds that have undergone biotic or abiotic transformation (humic substances). Although representing a small and variable, in time and space, fraction of SOM, DOM plays an integral role in the soil C cycle since it is claimed to regulate the mineralization of SOM and plant residues by co-metabolism and/or by triggering soil microbial biomass (SMB) into activity (Kuzyakov et al., 2000; De Nobili et al., 2001; Kemmitt et al., 2008). In addition, DOM can modulate soil nutrient cycles as it affects both the transport and microbial transformation of nitrogen (N), phosphorus, and sulphur (Zsolnay, 2003) as well as the availability of micronutrients, such as Fe and Zn (Cesco et al., 2000; Chen et al., 2004). From an environmental point of view, DOM represents a major source of dissolved C and nutrient losses in surface and subsurface waters: van Kessel et al. (2009) showed that up to 216 kg ha^−1^ year^−1^ of dissolved organic carbon (DOC) and 127 kg ha^−1^ year^−1^ of dissolved organic nitrogen (DON) can leach out of agricultural systems.

As the role of DOM is strictly regulated by its concentration and composition, the collection and sampling of undisturbed DOM is essential to obtain meaningful information. Chantigny et al. (2006) carried out a thorough review of methods, emphasizing that different approaches may result in the collection of different amounts and fractions of the soil solution.

Among DOM components, dissolved humic substances (DHS) have well-documented stimulatory effects on plant growth (Chen et al., 2004). The natural occurrence and role of humic substances in soils was questioned (Lehmann and Kleber, 2015) because of the harsh alkali-based procedures used for their extraction. However, the usefulness of the humic substances-based approach to understand natural organic matter processes has been recently confirmed (Olk et al., 2019). Furthermore, DHS can be obtained without the use of alkaline extractants by simply leaching soil with water. Treatment of plants with water-extractable humic substances from peat and vermicompost was shown to induce changes in root morphology and modulate nutrient acquisition, pathways of primary and secondary metabolism, and hormonal and reactive oxygen balance (Varanini and Pinton, 2001; Nardi et al., 2002; Zanin et al., 2019).

Numerous studies have been performed to understand the molecular mechanisms activated by plant exposure to humic substances. Varanini and Pinton (2001) distinguished between indirect effects (such as improved nutrient availability through metal binding) and direct effects. Among the latter, the improvement of root ion uptake capacity, and rhizosphere acidification *via* stimulation of plasma membrane H^+^-ATPase, and root proliferation involving hormone-like activity have been reported for humic substances (Varanini et al., 1993; Pinton et al., 1999; Canellas et al., 2002; Nardi et al., 2002; Zanin et al., 2015a; Zanin et al., 2015b; Zamboni et al., 2016). Transcriptomic studies indicated that root exposure to humic substances induced also changes in the expression profile of genes involved in the acquisition and assimilation of several nutrients, as shown in *Arabidopsis*, rapeseed, and maize (Trevisan et al., 2011; Jannin et al., 2012; Zanin et al., 2018). These effects depend on the origin, molecular size, and chemical characteristics of humic substances (Zandonadi et al., 2013; Olaetxea et al., 2018).

A frequent criticism raised by studies on the stimulatory activity of humic substances is that the investigations carried out so far have been implemented with humic substances extracted from organic-rich substrates (e.g., sphagnum peat, vermicompost, leonardite; Aguirre et al., 2009; Zanin et al., 2018) and none has actually employed DHS from cultivated mineral soils.

Poor information is also available on the chemical properties of DOM in soils under OF vs. CF and on the relationships between SOM and DOM in calcareous soils.

The aim of the present work was to investigate the biological properties and characteristics of DHS isolated from water leached from undisturbed soil monoliths of arable mineral soils. This approach allows avoiding any potential interference of the extraction procedure (Zsolnay, 2003).

While the conversion to OF can activate a positive trend towards the increase of SOM (Gattinger et al., 2012), no evidence for a similar trend has been noticed for DOM (Hu et al., 2018). Although OF is claimed to improve organic matter-related soil quality, evidence of the effects of OF on the amount and biological activity of humic substances is still lacking. In addition, while soil use and management have been recognized to have a significant impact on humic substances’ complexity and activities (Nardi et al., 2004; Olk et al., 2019), it is not known whether the presence of a crop can affect in itself the quality and quantity of DHS.

In this work, we investigated the biostimulant actions, on root development and nitrate acquisition by maize plants, of DHS isolated at different times of the year, from a CF and an OF soil, with and without the presence of plants.

To allow comparison with previous scientific literature and eventually validate the integrity of the use of water-extractable humic substances from organic soils, biological activities and chemical properties of DHS from the examined agricultural soils were compared with those of water-soluble humic substances extracted from peat (WEHS).

## Materials and Methods

### Soil Sampling and Monolith Column Setup

Soil samples were collected from two adjacent arable soils in Friuli Venezia Giulia Region (NE Italy). One site had been managed for 10 years according to OF (CE 2092/91, 834/07), while the other had been continuously managed with CF practices.

The soils examined were silty-loam Fluvisols with similar granulometric composition. Chemical and physical characteristics of the soils are given in **Table 1**. The pH (measured in water) of the two soils was alkaline and was even more alkaline in the OF soil, in agreement with its larger amount of active carbonate [7 vs. 2 g 100 g^−1^ dry weight (d.w.) in CF soil]. Both soils are characterized by low organic carbon (C_org_) and medium cation exchange capacity (CEC).

**Table 1:** Main physical and chemical traits of the soils under organic farming (OF) and conventional farming (CF).

Trait and method	Unit	OF	CF
Sand (>0.02 mm)	g 100 g^−1^ d.w.	22	18
Silt (0.02 ÷ 0.002 mm)	g 100 g^−1^ d.w.	62	58
Clay (<0.002 mm)	g 100 g^−1^ d.w.	16	24
Bulk density (excavation method)	Mg m^−3^	1.41	1.34
Saturated water content (0 MPa)	g 100 g^−1^ d.w.	37.5	40.4
Field capacity (−0.03 MPa)	g 100 g^−1^ d.w.	32.0	35.2
Permanent wilting point (−1.5 MPa)	g 100 g^−1^ d.w.	14.9	18.5
pH (H_2_O)		8.5	7.6
CEC (BaCl_2_, pH 8.2)	cmol^+^/kg d.w.	13.0	15.3
C_org_ (Walkley-Black)	g 100 g^−1^ d.w.	0.6	1.0
Active carbonates (Drouineau)	g 100 g^−1^ d.w.	7	2

**Figure 1:**
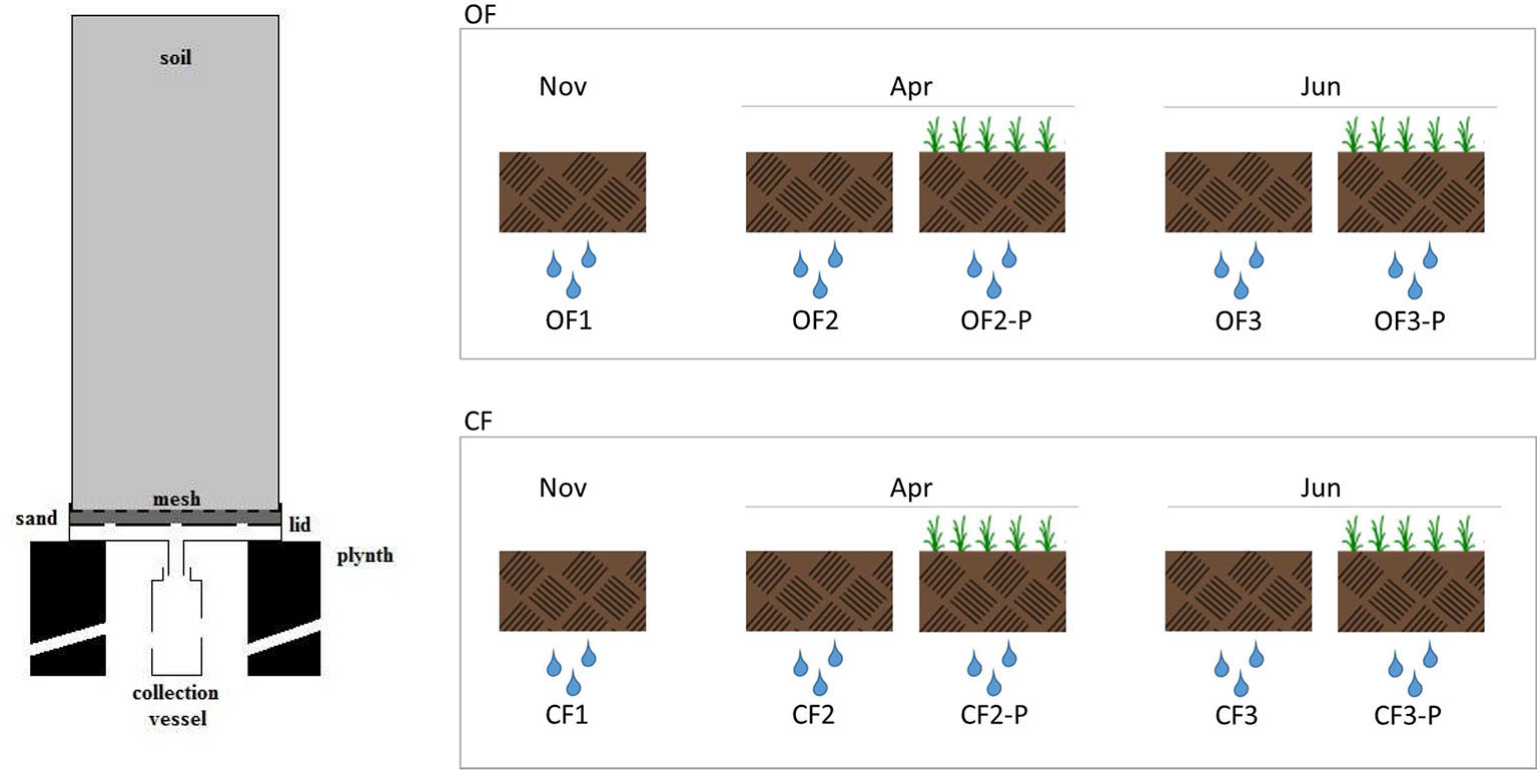
Cross-section of the soil column and leachate collection apparatus and experimental setup used in this study. The leachates were collected from organic farming soils (OF1, OF2, and OF3) or from conventional farming soils (CF1, CF2, and CF3). OF1 and CF1 were sampled in November; OF2, OF-P2, CF2, and CF-P2 were sampled in April; OF3, OF-P3, CF3, and CF-P3 were sampled in June. OF1, OF2, OF3 and CF1, CF2, CF3 refer to leachates collected from bare soil columns. OF-P2, OF-P3 and CF-P2, CF-3 refer to leachates collected from planted soil columns.

Undisturbed soil monolith columns were collected by gently driving polyvinyl chloride (PVC) pipes (30 cm internal diameter, 70 cm long) into the soil using a hydraulic press in order to reduce the impact on soil structure; soil water potential at sampling was about 0.6 ± 0.15 MPa. A trench was dug on one side to allow cutting the soil at the bottom of pipes and placing a nylon mesh to retain the soil; a perforated lid filled with coarse sand was finally welded before removal. Monolith columns were then arranged in a greenhouse following a completely randomized scheme and placed over concrete plinths, allowing the collection of leachates in PVC vessels (**Figure 1**). The experiment featured 200 monolith columns (100 with OF and 100 with CF soil) divided into two treatments: bare or planted with *Triticum aestivum* L., cv. Capo.

To collect DOM, monolith columns were subjected to three controlled drainage events: the first one was carried out in November, on bare soils, before seeding. The following events were carried out in April and June of the following year, corresponding, respectively, to the stem elongation (stage 3) and milk development (stage 7) of wheat plants (Zadoks et al., 1974). Each lysimeter was irrigated by suspended sprinklers providing about 15 mm/h, with a total of 1.4–1.7 L of water. Leachates were collected within 36 h and corresponding treatments were pooled together. The leachates collected from organic farming soils were called OF1, OF2, and OF3, while those collected from conventional farming soils were called CF1, CF2, and CF3. The leachates OF1 and CF1 were collected in November, OF2, OF-P2, CF2, and CF-P2 were sampled in April, and OF3, OF-P3, CF3, and CF-P3 were sampled in June. OF1, OF2, OF3 and CF1, CF2, CF3 refer to leachates collected from bare soil columns; OF-P2, OF-P3 and CF-P2, CF-P3 refer to leachates collected from planted soil columns (**Figure 1**).

The concentrations at field capacity (0.33 MPa) and wilting point (1.5 MPa) of soluble humic fractions in the soil solution were then calculated taking into account the hydrological properties of the soils and the recovered weight of DHA. In the sampling in November, the nitrate concentration in the leachates (before the DHS extraction) was about 14.9 mg L^−1^ in the OF and 24.5 mg L^−1^ in the CF soil.

### Isolation of DHS From Leachates

In order to isolate a sufficient amount of humic substances to carry out the plant growth and nitrate uptake experiments, leachates from replicate monoliths were pooled and 80 L of leachate was processed for each treatment. Leachates were, first of all, filtered on Whatman WCN 0.2-µm nitrocellulose membrane filters and then acidified to pH 1–2 with H_2_SO_4_ before being loaded onto SPE columns (400 mm × 30 mm) of cross-linked polyvinylpyrrolidone. Each column was washed with double-distilled water. Adsorbed DHS were then eluted with NaOH 0.1 M. The eluates were treated with Amberlite IR-120 (H^+^ from Sigma-Aldrich, Milan, Italy) to reduce ash content, adjusted to neutrality with KOH 0.1 M, and freeze-dried for storage before further analyses.

### Isolation of Humic Substances From Sphagnum Peat (WEHS)

The WEHS were obtained as previously reported by Tomasi et al. (2009). Briefly, 50 ml of distilled water was added to 2.5 g of sphagnum peat (Novobalt, Lithuania) and shaken for 15 h at room temperature. The solution was filtered through a Whatman WCN 0.2-μm membrane filter and acidified to pH 1–2 with H_2_SO_4_. To concentrate and purify humic substances, the solution was loaded onto an Amberlite XAD-8 column (Ø 20 mm, height 200 mm; Sigma-Aldrich, Milan, Italy; Aiken et al., 1979). The column was washed with 100 ml of distilled water and the adsorbed humic substances eluted with 0.1 M NaOH. To remove exchangeable metals, WEHS were treated with Amberlite IR-120 H^+^ from Sigma-Aldrich (Milan, Italy) and then adjusted to neutrality with 0.1 M NaOH. WEHS were stored as freeze-dried powder and redissolved in distilled water before use. Characterization of WEHS was reported by Tomasi et al. (2013).

### Chemical Characterization of DHS

Molecular weight (MW) distributions were determined by high-performance liquid–size exclusion chromatography (HPLC-SEC) with a Bio-Rad Bio-Sil SEC 250-5 column (300 mm × 7.8 mm) and a Waters 484 Millipore UV–visible detector. The elution was performed with a 75-mM TRIS-phosphate buffer at pH 7.5 and column calibrated with a set of polystyrene sulfonate standards. Freeze-dried DHS samples were first dissolved into the TRIS-phosphate buffer at a concentration of 2 mg/ml and filtered with Minisart filters (0.20 µm). Afterwards, 20 µl of each sample was injected through a loop system into the flux of the eluting solution. The elaboration of the chromatogram obtained by recording absorbance at 400 nm allowed calculation of their molecular weight distribution.

*E*_465_/*E*_665_ ratios were calculated from absorbances measured at 465 and 665 nm on 2 mg ml^−1^ DHS in 75 mM sodium bicarbonate buffer (pH 7.5).

Estimation of the number of carboxylic functional groups was performed using a Mettler Toledo titrator DL50 version 2.4. Freeze-dried DHS samples were dissolved in ultra-deionized deaerated Milli Q water to obtain a sample concentration of 4 mg/ml. Solutions were acidified to about pH 2 with Amberlite IR-120^+^ and 4 ml aliquots were titrated under N_2_ by addition of 0.05 ml of NaOH 0.1 M with an equilibration time of 2 min up to a maximum volume of 1.5 ml of the titrant.

Fourier transform infrared (FTIR) spectra of freeze-dried DHS (pH 7) were recorded from 4,000 to 700 cm^−1^ at a resolution of 4 cm^−1^ on KBr pellets. About 2–3 mg of oven-dried humic sample and anhydrous KBr powder (both dried for 24 h at 105°C) were mixed together, ground, and hydraulically pressed into 1-mm-thick pellets.

### Plant Growth for Experiments With DHS

Maize plants (*Zea mays* L., PR33T56, Pioneer Hybrid Italia S.p.A.) were hydroponically grown as previously described by Zanin et al. (2018). Therefore, after germination over aerated 0.5 mM CaSO_4_ solution, maize seedlings (3 days old) were transferred into an aerated hydroponic system under controlled conditions (16/8-h light/dark cycle, 220 µmol m^−2^ s^−1^ light intensity, 25/20°C temperature, 70–80% relative humidity). After 2 days, maize plants (5 days old) were transferred to a N-free nutrient solution (in μM: CaSO_4_, 500; KH_2_PO_4_, 175; MgSO_4_, 100; NaFe-EDTA, 20; KCl, 5; H_3_BO_3_, 2.5; MnSO_4_, 0.2; ZnSO_4_, 0.2; CuSO_4_, 0.05; Na_2_MoO_4_, 0.05).

After 1 h from the beginning of the light phase, nitrogen was added to nutrient solution in the form of calcium nitrate, 0.5 mM Ca(NO_3_)_2_, with or without 5 mg C_org_ L^−1^ of isolated humic substances (DHS or WEHS) as described by Pinton et al. (1999). The pH of solution was adjusted to pH 6.0 using potassium hydroxide.

The treatments lasted up to 24 h (for physiological and molecular analyses); during this time, plants were harvested and used for the analyses described below.

### Measurement of Net High-Affinity Nitrate Uptake

The net influx of nitrate into roots of maize seedling was evaluated by depletion from an assay solution containing 0.2 mM KNO_3_ and 0.5 mM CaSO_4_, as described by Pinton et al. (1999). Briefly, maize seedlings were washed in 0.5 mM CaSO_4_ and roots were incubated for 10 min in the assay solution. The assay solution was sampled (0.2 ml) every 2 min and mixed thoroughly with 0.8 ml of 5% (*w*/*v*) salicylic acid in concentrated H_2_SO_4_. After 20 min incubation at room temperature, 19 ml of 2 M NaOH was added to each sample. Samples were cooled to room temperature and nitrate concentrations were determined spectrophotometrically at 410 nm, as described by Cataldo et al. (1975). The net uptake rate was expressed as micromoles of nitrate per gram of root fresh weight (FW) per hour.

### Real-Time RT-PCR Analyses

Real-time reverse transcription PCR (RT-PCR) analyses were performed as described by Zanin et al. (2016). Using Primer3 software (Koressaar and Remm, 2007; Untergrasser et al., 2012), primers were designed and synthesized by Sigma-Aldrich (**Supplementary Table S1**). The analyses were performed using the Opticon Monitor 2 software (Bio-Rad) and qPCR package for statistical R software (R version 2.9.0; www.dr-spiess.de/qpcR.html). For each primer, efficiencies of amplification were determined as indicated by Spiess et al. (2008). Three reference genes (*ZmRPL17*, *ZmGADPH*, and *ZmTUA*) were used to normalize the real-time RT-PCR data. Data were normalized using the 2^–ΔΔCT^ method (Livak and Schmittgen, 2001).

### Statistical Analyses

Physiological and transcriptional analyses were performed on three independent biological replicates obtained from independent experiments (*N* = 3); a pool of six plants was used for each sample. Statistical significance was determined by one-way analysis of variance (ANOVA) using Holm–Sidak test (*p* < 0.05, *N* = 3). Statistical analyses were performed using SigmaPlot version 12.0 software.

DHS were isolated from pooled leachates of 50 monolith columns (80 L of pooled leachate for each treatment). Therefore, no statistical treatment of results was carried out and the reported standard deviation refers to the analytical variability of each measurement.

## Results

### Biological Action of DHS

**Figure 2:**
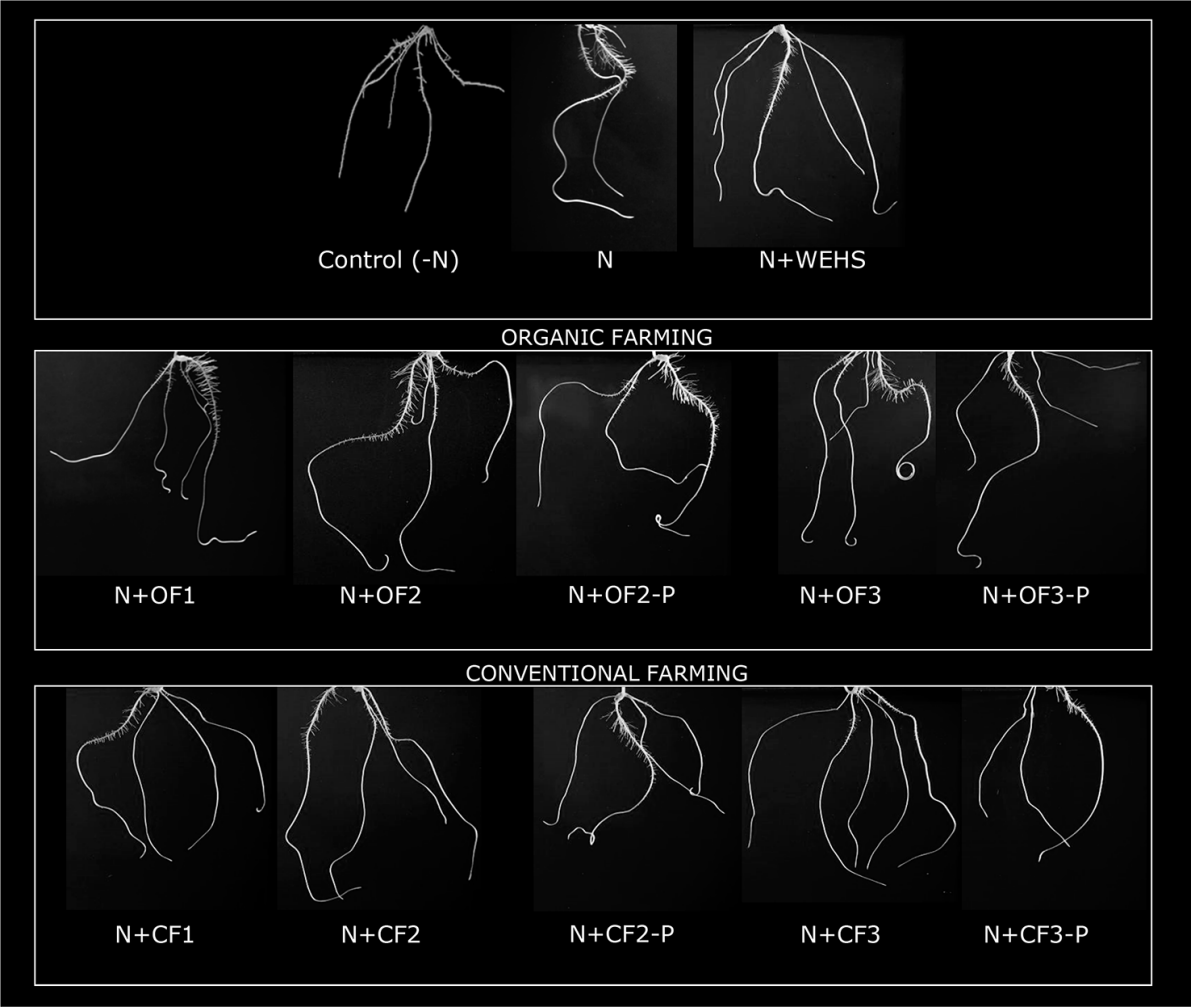
Representative pictures of whole root system of maize plants (5 days old) after 24 h of treatment (in nutrient solution): N-free nutrient solution [*Control (-N)*], nutrient solution containing nitrate (0.5 mM Ca(NO_3_)_2_) with or without 5 mg C_org_ L^−1^ water-extractable humic substances (WEHS) (humic substances isolated from sphagnum peat; *N+WEHS* or *N*, respectively), nitrate (0.5 mM Ca(NO_3_)_2_) with dissolved humic substances (DHS) (5 mg C_org_ L^−1^) isolated from organic farming (OF) soils (*N+OF1*, *N+OF2*, *N+OF-P2*, *N+OF3*, and *N+OF-P3*) or isolated from conventional farming (CF) soils (*N+CF1*, *N+CF2*, *N+CF-P2*, *N+CF3*, and *N+CF-P3*). The code name of samples is reported in **Figure 1**.

The biological activity of DHS isolated from organic farming soil or from conventional farming soils (OF or CF soils, respectively) were tested on maize plants after adding 5 mg C_org_ L^−1^ DHS to nutrient solution. As positive control, WEHS were used adding 5 mg C_org_ L^−1^ to nutrient solution. After 24 h, no significant changes in root growth were visible in WEHS-treated plants (N+WEHS; **Figure 2**) in comparison to control plants (N). On the contrary, DHS promoted visible root elongation and proliferation already after 24 h of treatment. Depending on their origin, some differences on the elongation and number of secondary roots were observed in the stimulatory effect of DHS. Plants treated with DHS isolated from OF soils induced a larger proliferation of secondary roots (N+OF1, N+OF2, and N+OF3; **Figure 2**). Moreover, the stimulatory action varied with sampling time as plants treated with DHS leached in June and particularly those leached from the CF soil (N+CF3) showed a lower capability to stimulate proliferation of secondary roots.

**Figure 3:**
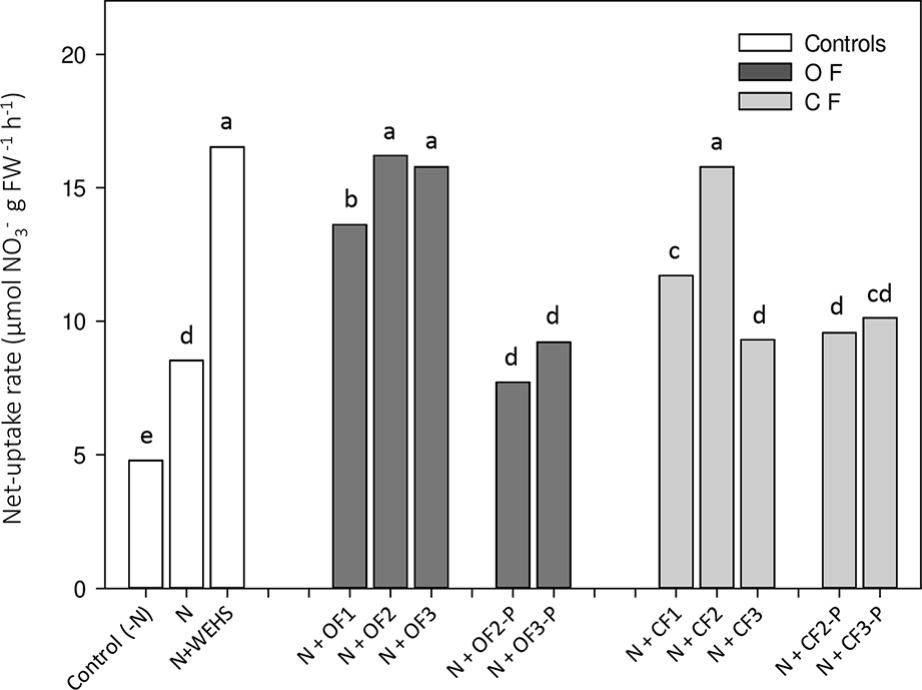
Net uptake rates of nitrate by roots of maize plants exposed for 4 h to humic substances of different origin. *White bars*, As controls, plants exposed to N-free nutrient solution [*Control (-N)*, or nutrient solution containing nitrate (0.5 mM Ca(NO_3_)_2_) with or without water-extractable humic substances (WEHS) (5 mg C_org_ L^−1^; *N+WEHS* or *N*, respectively). *Dark gray bars*, Plants exposed to nutrient solution with nitrate and dissolved humic substances (DHS) (5 mg C_org_ L^−1^) isolated from organic farming (OF) soils. *Light gray bars*, Plants exposed to nutrient solution with nitrate and DHS (5 mg C_org_ L^−1^) isolated from conventional farming (CF) soils. The code name of samples is reported in **Figure 1**. Bars with the same letters are not significantly different at *p* ≤ 0.05.

Net uptake rates of nitrate were measured on whole root systems of maize plants. After 4 h of treatment, WEHS (N+WEHS plants; **Figure 3**) promoted nitrate acquisition, doubling the capability of maize roots to take up nitrate in comparison to nitrate-treated control plants (N plants). Also, DHS isolated in autumn and spring from bare soil leachates of OF and CF soils were able to enhance the net nitrate uptake rates after 4 h (N+OF1, N+OF2, N+CF1, and N+CF2; **Figure 3**). The stimulatory effect on root nitrate uptake was also evident following application of DHS collected in June from OF bare soils (N+OF3), but DHS collected in June from CF soils did not increase the capability of plants to take up nitrate (N+CF3). DHS extracted from planted soils, irrespectively to soil management, had no stimulatory effect on root nitrate uptake (N+OF-P2, N+OFP3, N+CF-P2, and N+CFP3; **Figure 3**).

**Figure 4:**
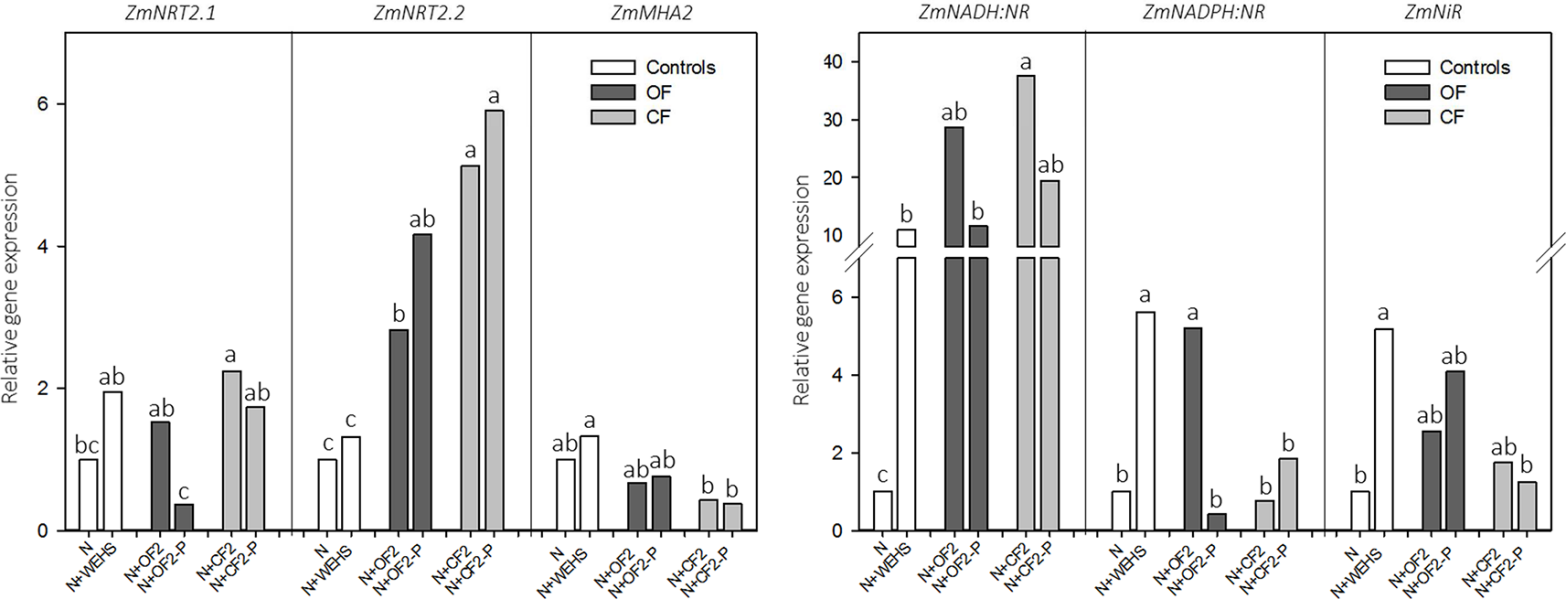
Real-time RT-PCR analyses of the main genes involved in the nitrate acquisition—*ZmNRT2.1* and *ZmNRT2.2* (coding for two high-affinity nitrate transporters), *ZmMHA2* (coding for a PM H^+^-proton pump), *ZmNADPH:NR* and *ZmNADPH:NR* (coding for two isoforms of nitrate reductase), and *ZmNiR* (coding for nitrite reductase)—and performed on roots of maize plants exposed for 2 h to humic substances of different origin isolated in April. *White bars*, As controls, plants exposed to nutrient solution containing nitrate (0.5 mM Ca(NO_3_)_2_) with or without water-extractable humic substances (WEHS) (5 mg C_org_ L^−1^) (*N+WEHS* or *N*, respectively). *Dark gray bars*, Plants exposed to nutrient solution with nitrate and dissolved humic substances (DHS) (5 mg C_org_ L^−1^) isolated from organic farming (OF) soils. *Light gray bars*, Plants exposed to nutrient solution with nitrate and DHS (5 mg C_org_ L^−1^) isolated from conventional farming (CF) soils. The code name of samples is reported in **Figure 1**. Bars with the same letters are not significantly different at *p* ≤ 0.05.

To validate the physiological response of plants to DHS exposure, real-time RT-PCR analyses were performed on those genes most involved in nitrate acquisition, as *ZmNRT2.1*, *ZmNRT2.2*, and *ZmMHA2* (coding for two high-affinity nitrate transporters and a PM H^+^-proton pump) and *ZmNADH:NR*, *ZmNADPH:NR*, and *ZmNiR* (coding for assimilatory enzymes, as two isoforms of nitrate reductase and nitrite reductase; **Figure 4**). The analyses were performed on maize roots treated with DHS isolated in April (OF2, OF-P2, CF2, and CF-P2), which induced the maximum uptake rate of nitrate. After 2 h of treatment, the expression in maize roots of *ZmNRT2.1*, *ZmNRT2.2*, and *ZmMHA2* did not respond to treatment with WEHS. On the other hand, all DHS induced the upregulation of *ZmNRT2.2*, and DHS isolated in April from not planted CF soil (N+CF2; **Figure 4**) induced also the upregulation of *ZmNRT2.1* in comparison to control plants (N). Plants treated with DHS did not alter significantly the expression of *ZmMHA2*, although a slight reduction of its expression occurred in the presence of CF DHS (CF2 and CF-P2; **Figure 4**).

Concerning the nitrate reductive pathway, the treatment with WEHS upregulated the transcripts encoding nitrate and nitrite reductases; in particular, the expressions of *ZmNADH:NR*, *ZmNADPH:NR*, and *ZmNiR* were at least five times higher than the expression levels induced by nitrate alone. A significant upregulation of *ZmNADPH:NR* was induced also by all tested DHS fractions in comparison to nitrate only, while the upregulation of *ZmNADPH:NR* occurred only with the N+OF2 treatment. In comparison to the control (N treatment), no significant changes in the expression of *ZmNiR* were caused by the treatment with DHS (N+OF2, N+OF-P2, N+CF2, and N+CF-P2).

### Quantitative and Chemical Characteristics of DHS in CF and OF Leachates

The concentration range of soluble humic carbon in the soil solution of the two soils, calculated by dividing the total DHS carbon of leachates by the water content of soil monoliths at field capacity and wilting point, ranged between 5.3 and 27.1 mg C_org_ L^−1^ (**Table 2**).

**Table 2:** Estimated concentration ranges (mg C_org_ L^−1^) of soluble humic fractions (dissolved humic substances, DHS) in the soil solution of bare soils (OF and CF) and planted soils (OF-P and CF-P) at field capacity and wilting point.

	OF	OF-P	CF	CF-P
DHS at field capacity (mg C_org_ L^−1^)				
November	10.5		10.8	
April	11.9	8.3	8.8	5.3
June	6.3	8.5	14.3	11.7
*Mean value	9.1		10.2	
	**OF**	**OF-P**	**CF**	**CF-P**
**DHS at wilting point (mg C_org_ L^−1^)**				
November	22.3		20.4	
April	25.4	17.7	16.7	10.0
June	13.4	18.1	27.1	22.1
*Mean value	19.4		19.3	

*Mean values refer to all soil treatments and sampling times (N = 5).

At field capacity, DHS concentrations were slightly higher in CF with respect to OF soils (mean values: 10.2 vs. 9.1 mg C_org_ L^−1^) and in bare compared to planted soils (mean values: 10.4 vs. 8.4 mg C_org_ L^−1^).

**Figure 5:**
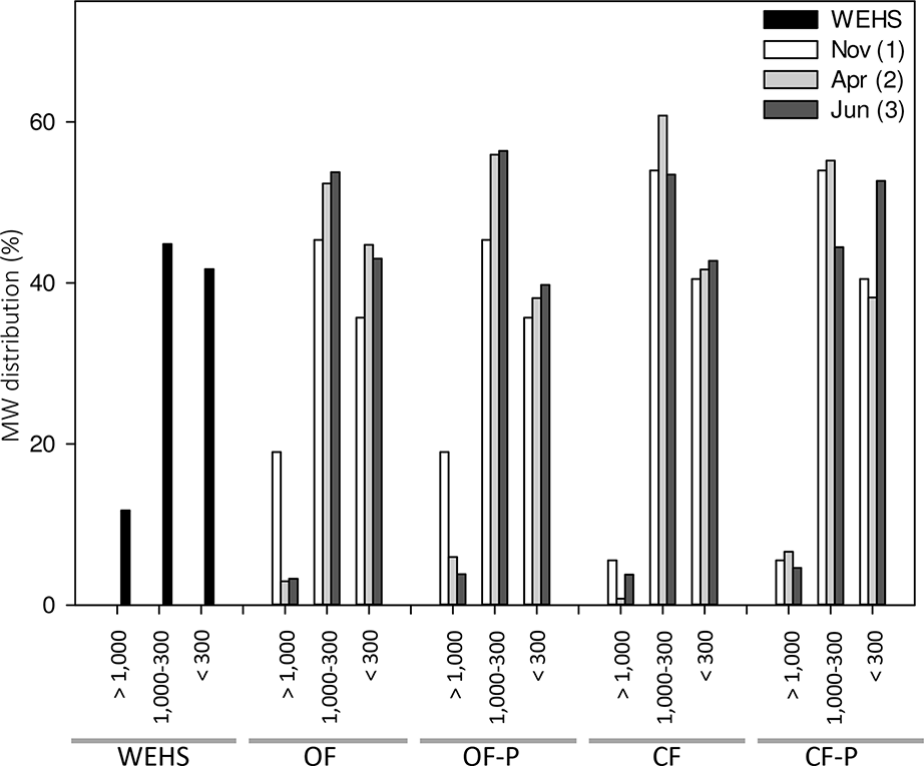
Molecular weight (MW expressed in dalton) distribution of humic substances isolated from leachates collected from bare and planted soils at different sampling times. As control, MW distribution of water-extractable humic substances (WEHS) isolated from sphagnum peat (Novobalt, Lithuania) are also shown. *Nov (1)* refers to leachates sampled in November; *Apr (2)* refers to leachates sampled in April; *Jun (3)* refers to leachates sampled in June. *OF* and *CF* refer to leachates collected from bare soil columns; *OF-P* and *CF-P* refer to leachates collected from planted soil columns.

DHS are expected to be mostly composed of small molecular size components. Size exclusion chromatography of DHS (**Figure 5**) confirmed this assumption, but showed that a fraction of relatively large molecules (e.g., apparent MW > 1,000 Da) was present at the beginning of the experiment (November) and particularly in the OF soil (18% of large molecules in OF1). The molecular size distribution of DHS isolated in November from the OF soil was the most similar to that of the WHSH from peat.

However, in leachates collected from the same soil in April (OF2) and June (OF3), only small amounts of high apparent MW components occurred and the percentage of DHS with an apparent MW < 1,000 Da increased. Fractions of apparent MW between 1,000 and 300 Da were more abundant in planted soils (OF-P2 and OF-P3).

In the DHS from the CF soil, apparent MW fractions between 1,000 and 300 Da accounted for 45–60%, and the smallest molecules (apparent MW < 300 Da) accounted for 40–50% of the total DHS. In particular, the percentage of substances with apparent size between 1,000 and 300 Da was largest in bare soil leachates collected in April (CF2) and lowest in planted soil leachates collected in June (CF-P3); the latter also showed the highest enrichment of very low MW fraction (< 300 Da apparent MW).

**Figure 6:**
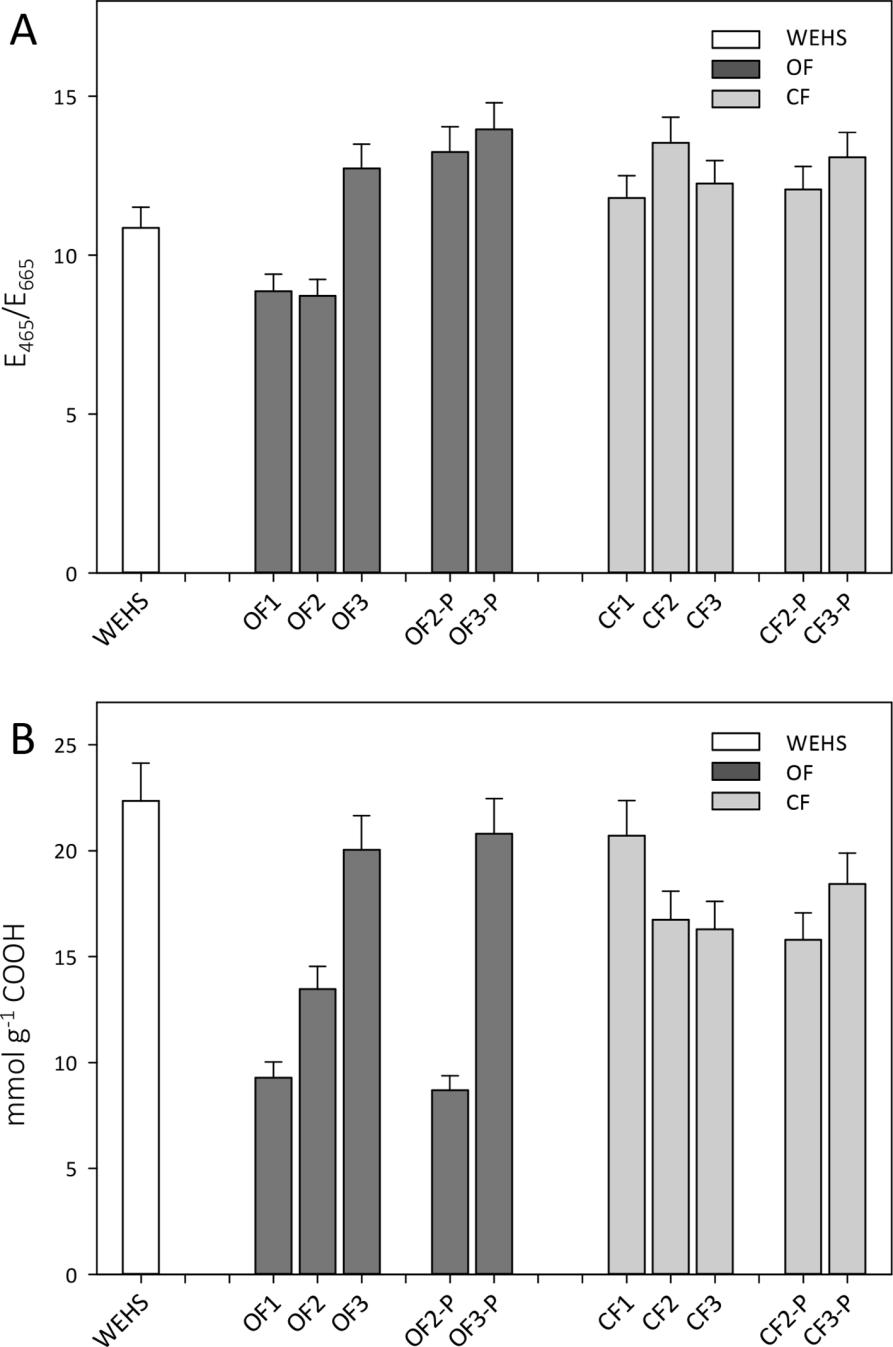
*E*_465_/*E*_665_ ratios of organic farming (OF) and conventional farming (CF) fractions **(A)** and density of carboxylic groups in dissolved humic substances (DHS) obtained by titration with NaOH 0.1 M **(B)**. *White bar* refers to water-extractable humic substances (WEHS); *dark gray bars* refer to DHS isolated from OF soils; *light gray bars* refer to DHS isolated from CF soils (data shown are means plus standard deviation). OF1 and CF1 were sampled in November; OF2, OF-P2, CF2, and CF-P2 were sampled in April; OF3, OF-P3, CF3, and CF-P3 were sampled in June. OF1, OF2, OF3 and CF1, CF2, CF3 refer to leachates collected from bare soil columns. OF-P2, OF-P3 and CF-P2, CF-P3 refer to leachates collected from planted soil columns.

Trend observed by size exclusion chromatography were confirmed by *E*_465_/*E*_665_ absorption ratios which are inversely related to the molecular size of HS. The *E*_465_/*E*_665_ values were typical of small-sized HS, i.e., fulvic acids, and relatively lower in OF soils in November (8.9, OF1). *E*_465_/*E*_665_ ratios increased during the experiment (OF2 and OF3), especially in planted soils (OF-P3; **Figure 6A**). In June, the *E*_465_/*E*_665_ of bare OF soils was 12.7 (OF3), while in planted OF soils it reached a value of 14 (OF-P3). In the CF soil, DHS had larger and more constant *E*_465_/*E*_665_ ratios, namely, 11.8 in November (CF1) and respectively 12.2 and 13.1 in June in bare and planted soils (CF3 and CF-P3).

The content of carboxylic groups (**Figure 6B**) increased steadily throughout the experiment in the DHS of the OF soil, whereas it decreased in DHS leached from the CF soil. In November, the total density of carboxylic groups in DHS of the OF soil was about half than that observed in the CF soil (9.3 mmol g^−1^ in OF1 vs. 20.7 mmol g^−1^ in CF1), but during the experiment the amount of carboxylic groups increased in the OF DHS. In June, the amount of carboxylic groups in OF soils was around 20 mmol g^−1^ of DHS independently of the plant presence (OF3 and OF-P3). In CF soil, DHS showed a slight decrease in carboxyl content during the time of the experiment for both bare and planted soils, as in summer the amounts of carboxylic groups were 16.3 mmol g^−1^ (in CF3) and 18.4 mmol g^−1^ (in CF-P3), respectively.

**Figure 7:**
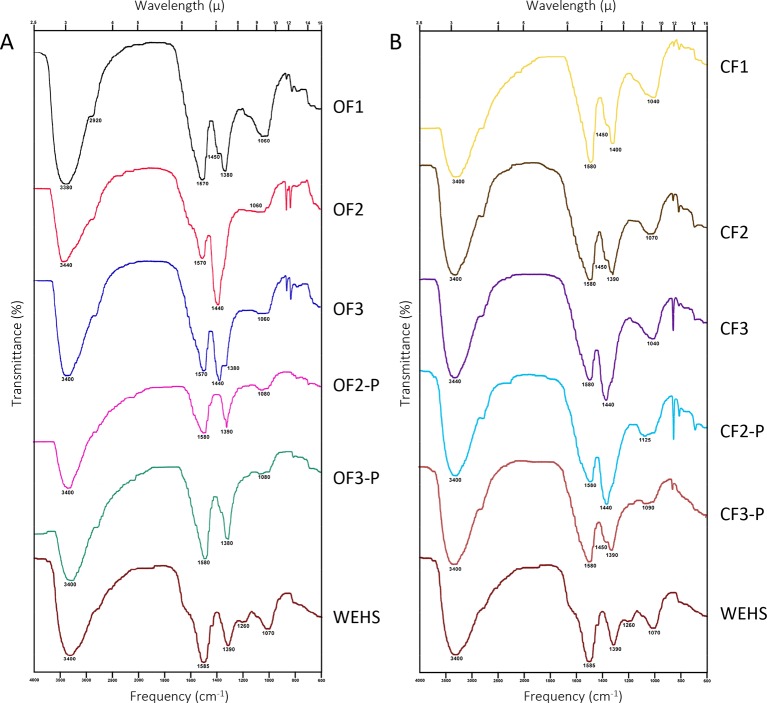
FTIR spectra of dissolved humic substances (DHS) fractions isolated from organic farming (OF) soils **(A)** and from conventional farming (CF) soils **(B)**. OF1 and CF1 were sampled in November; OF2, OF-P2, CF2, and CF-P2 were sampled in April; OF3, OF-P3, CF3, and CF-P3 were sampled in June. OF1, OF2, OF3 and CF1, CF2, CF3 refer to leachates collected from bare soil columns. OF-P2, OF-P3 and CF-P2, CF-P3 refer to leachates collected from planted soil columns.

The characterization of DHS was further achieved by FTIR spectroscopy (**Figure 7**). Compared with most FTIR spectra of HS, DHS and WEHS spectra are relatively simple and characterized by four main bands. All spectra displayed intense very broad absorption in the region between 3,440 and 3,380 cm^−1^. This band corresponds to O–H stretching vibrations of phenolic groups overimposed on O–H stretching of carbohydrates (Coates, 2006). The intensity of this band was more pronounced in DHS of both soils at the beginning of the experiment (OF1 and CF1). In the organically managed soil, it shifted to lower wavelengths (3,440–3,400 cm^−1^) and became broader with time, indicating stronger hydration and H bonding, but also an increasing contribution from the stretching vibration of O–H in phenols. The progressively lower presence of carbohydrate moieties in DHS molecules from the OF soil is confirmed by the decrease of absorbance around 1,080–1,040 cm^−1^. The weak broad absorption around 1,080–1,040 cm^−1^ may, in fact, be assigned to C–O and C–C stretching vibrations of carbohydrate rings. In OF-leached DHS, this band decreased during the experiment, whereas it remained stable till June in CF-leached DHS.

All spectra also exhibited very weak absorption due to aliphatic C–H stretching at about 2,920 cm^−1^.

Strong asymmetric and symmetric COO^−^ stretching bands, coherent with the fact that DHS were freeze-dried at pH 7, are present in all spectra. These bands are located around 1,570 and 1,440 cm^−1^ in the DHS of organically managed soil at the beginning of the experiment (OF1). The first band shifts to longer wavelengths (1,590 cm^−1^) towards the end of the experiment (OF3 and OF3-P), which may indicate loss of double bonds conjugated to carboxyl groups. Conversely, in all DHS from CF soil, the band remains centered at 1,580 cm^−1^.

Besides symmetric COO^−^ stretching, bands in the 1,440–1,380 region can also be attributed to the absorption of C–O groups in phenols and tertiary alcohols. The shift of maximum intensity from the 1,440 to the 1,380 band, which contributes to absorption in this region, is related to a stronger contribution of this type of structures.

In fact, the ratio between the absorbance intensity of the two main peaks is related to asymmetric and symmetric COO^−^ stretching at respectively 1,590–1,570 cm^−1^ and 1,440–1,380 cm^−1^. If all absorption in this region was due to carboxylate moieties, the value of this ratio would be about 1.4 (Max and Chapados, 2004). Under both types of soil management, DHS collected in November showed ratios (1.34 in OF1 soil and 1.38 in CF1 soil) compatible with a nearly exclusive contribution from carboxyls. In April, however, all samples showed a ratio lower than 1, with the only exception of the bare CF soil that maintained a ratio of 1.29 (CF2). Independently from management, DHS collected in June from planted soils exhibited again high values of the ratio (1.41 in OF-P3 soil and 1.50 in CF-P3 soil), indicating release of carboxylic and polycarboxylic substances. Conversely, in the absence of plants, ratios remained lower than 1 in OF3 and CF3.

## Discussion

In the present work, DHS were extracted from leachates of soil monoliths through a procedure that mimics the natural process of “extraction” of humic substances by rainwater percolating through soil horizons under field conditions (Olaetxea et al., 2018). The two soils selected were cultivated soils of low and comparable organic matter content, which are highly representative of real agricultural field conditions. These soils are sub-alkaline soils rich in calcium carbonate, which strongly suppresses solubility of HS. Our results therefore demonstrate, first of all, that even in arable calcareous soils of low organic matter content some humic substances are dissolved in the soil solution and can therefore act in agricultural soils as they do in hydroponic experiments. Another important issue is the concentration of DHS: the minimum calculated DHS concentration (field capacity) was about 10 mg C_org_ L^−1^ in both soils and slightly larger in bare than in planted soils (mean values: 10.4 mg C_org_ L^−1^ vs. 8.4 mg C_org_ L^−1^). These concentration ranges represent a good approximation of the actual concentration of DHS in the solution of cultivated soils. It is important to underline that these concentrations are even larger than those usually applied in biological tests (**Table 2**) (Pinton et al., 1999; Cesco et al., 2000; Zanin et al., 2019), which are therefore validated by our results from a quantitative point of view.

Over time, leachates of OF soil showed an overall reduction in DHS content (**Table 2**). In the bare OF soil, the stronger decrease was observed between April and June (OF2 and OF3), while the presence of plants stabilized DHS concentrations in leachates (OF-P2 and OF-P3). A different behavior was observed in CF soil leachates since in this soil DHS concentrations were smallest in April (CF2 and CF-P2) and increased in June (CF3 and CF-P3), irrespectively of the presence of plants.

At the morphological level, the presence of DHS induced an overall higher development and proliferation of secondary and lateral roots in maize plants, confirming the biostimulant action of humic substances on plant growth (Canellas et al., 2002; Nardi et al., 2002).

Previous works reported a direct effect of WEHS on roots promoting nitrate acquisition. Like WEHS (Zanin et al., 2018), DHS also promoted nitrate uptake in roots. However, their action changed depending on time of the year and type of soil management and was nullified in the presence of growing plants. After 4 h of root contact with nitrate and DHS, an overall larger and more stable biostimulatory effect was observed with DHS from OF soils (N+OF1, N+OF2, and N+OF3). When maize plants were treated with DHS deriving from CF soils, nitrate uptake rates were highly variable, and the biostimulant effect occurred only with DHS collected in April, N+CF2 (a significant but mild effect was observed in November, N+CF1).

Transcriptional analyses of the genes most involved in N acquisition highlighted changes in their expression patterns which depended on the nature of DHS.

Confirming previous results reported in literature (Zanin et al., 2018), WEHS stimulated root expression of transcripts coding for N assimilatory enzymes more than for N transporters. Indeed, no significant changes in the expression levels of *ZmNRT2.1*, *ZmNRT2.2*, and *ZmMHA2* occurred between nitrate-treated plants and those treated with nitrate plus WEHS (N vs. N+WEHS). In contrast, our results indicate that the addition of DHS to nitrate-containing nutrient solution significantly promoted the expression of *ZmNRT2s* high-affinity nitrate transporters. In particular, in comparison to OF, the CF soil management results in the production of DHS that enhance the expression of both nitrate transporters, *ZmNRT2.1* and *ZmNRT2.2*. Besides transcriptional regulation, it has been reported that, in maize, the uptake rate of nitrate is also regulated at translational level, based on protein–protein interactions of NRTs and accessory protein (NRT3.1; Pii et al., 2016). Therefore, it is plausible to suppose that the biostimulant action exerted by humic substances might be ascribed to a stimulation at transcriptional level in root cells and also to a modulation of the interactions between proteins on the plasma membrane of root cells (e.g., nitrate transporters and proton pumps).

Concerning nitrate assimilation, initial reductive reactions are key points of this pathway and are mediated by nitrate and nitrite reductases (Nacry et al., 2013). Two isoforms of this enzyme are ubiquitously expressed in maize roots (Pandey et al., 1997): one is NADH-dependent (E.C. 1.6.6.1) and the other NAD(P)H-dependent (E.C. 1.6.6.2). The bispecific NAD(P)H:NR isoform occurs in many species (Srivastava, 1992), including roots and scutellum of maize seedlings, but not leaves (Redinbaugh and Campbell, 1981). However, their physiological role and their specific contribution to N assimilation are still unclear. As reported above, the treatment with WEHS induced the expression of transcripts encoding nitrate and nitrite reductases. Similarly, all tested DHS fractions also induced the upregulation of NR, and in particular the OF2 DHS stimulated the expression of both tested transcripts encoding for two NR isoforms.

This evidence might indicate that the isolated humic substances exerted the same stimulatory effect on nitrate acquisition but, depending on their origin (soil or peat), this physiological response might be acting on the expression of different molecular components. High-affinity nitrate transporters and nitrate reductase are activated by soil DHS, whereas peat WEHS act mainly on assimilatory enzymes.

Within the same agricultural management (CF or OF), gene expression analyses showed only slight variations among treatments. However, the stable stimulatory action of OF DHS on nitrate uptake rates (from November to June) might be a consequence of a wider and more active upregulation of molecular components involved in nitrate acquisition (nitrate transporters and reductive enzymes), while CF DHS induced the expression of only one isoform of nitrate reductase (*ZmNADH:NR*).

These differences can only in part be justified on the basis of changes in chemical characteristics recorded in the collected DHS. DHS leached from the two soils differ from peat WEHS. The apparent MW distribution of DHS showed that components with apparent MW > 1,000 Da (which are a sizeable fraction of WEHS) were present only in the OF soil. This fraction might be associated with abundance of organic C inputs relative to C mineralization, such as occurs in peat and in soils which receive organic amendments. Coherently with this hypothesis, this fraction strongly diminished during the experiment since no organic fertilizer or amendment was applied. This is also in agreement with the increasing oxidation observed in OF DHS (increased number of carboxyl groups and reduced structural contribution of carbohydrates).

High-MW components are obviously lacking in the CF soil that has not received organic amendments for a long time. At the beginning of the experiment in November, CF leachates already had a very low content of DHS with high apparent MW (CF1), and this fraction did not decrease in June.

The overall trend of molecular weight distributions is confirmed by the trend of *E*_465_/*E*_665_ absorption ratios which are also linked to the average molecular size of humic substances (Chen et al., 1977).

Besides their low apparent molecular sizes and coherently with their solubility, DHS fractions were characterized also by a high content of carboxylic functional groups. During time, the CF DHS showed a large and stable density of COOH, which was altogether quite similar to that of the WEHS fraction. A wider variability was recorded in OF DHS since the COOH content reached values similar to those recorded for WEHS only at the end of the treatment (June, OF3), while at the beginning of the experiment (November, OF1) the COOH content was about 50% lower. During the experiment, the increase in the carboxylic group content, in the *E*_465_/*E*_665_ ratio, and the prevalence of smaller molecules, as well as trends of absorption of oxygen containing functional groups in FTIR spectra, indicated that the DHS fractions underwent fragmentation and oxidation.

Before isolation of DHS, all leachates were analyzed also for their nitrate content. The nitrate leached from CF soil was twice that collected from the OF soil. This behavior might be a direct consequence of the agricultural management of soil and, indirectly, a consequence of a different rate of nitrification processes occurring thereafter in the OF and CF monolith columns. This latter hypothesis is in agreement with the FTIR spectra that displayed a decrease of the carbohydrate C–O stretching signal (1,040 cm^−1^) in the OF DHS fractions in both planted and non-planted soils during the experiment, suggesting the occurrence of extensive organic matter mineralization from November to June. Decomposition of carbohydrates might have been accompanied by an overall immobilization of mineral N in microbial cells. In the non-planted CF soil (CF1, CF2, and CF3), the FTIR spectra showed a much lower decrease of the C–O stretching signal: it is likely that the microbial biomass was less active in this treatment than in the OF soil, and therefore more nitrogen (in the form of nitrate) was present in leacheates from the bare CF soil. The same happened in the planted CF soil (CF-P2 and CF-P3), but wheat plants appeared to support mineralization, as shown by the decrease of the C–O stretching signal, which became comparable to that observed in the OF soil.

It is interesting to observe that DHS from planted soils exerted a weak effect on maize, and in particular did not display the capability to stimulate the nitrate acquisition. Two hypotheses can be formulated to explain this effect. In the first place, it is possible that the presence of wheat roots might have boosted biological activity and stimulated mineralization in the rhizosphere, leading to modification or decomposition of bioactive components of humic substances fraction. This hypothesis is supported by the FTIR spectra of planted CF and OF DHS, which showed distinct changes in absorbance intensity ratios between the two main peaks of carboxylate ions related to antisymmetric and symmetric COO^−^ stretching. This pattern, however, may also support the hypothesis that some aromatic compounds, such as phenols and flavonoids, may have been released by wheat roots. Flavonoids or similar compounds might have been sorbed on the PVP resin, together with humic substances. In literature, it is widely reported that phenolic compounds are the major secondary metabolites involved in plant allelopathy (Li et al., 2010) and might therefore impact nutrient acquisition in other crop species. On the other hand, the FTIR spectra and the analysis of carboxyl groups of DHS also bear evidence of extensive oxidation of organic matter in soil monoliths, which may have resulted in DHS with a low capability to stimulate nitrate acquisition.

## Conclusion

This study showed that OF and CF managements of soil qualitatively modify the characteristics and biostimulant potential of DHS and that the presence of plant roots also resulted in a dynamic interaction with these active components of the soil solution.

Further studies will be necessary to find out whether modification of DHS composition or their enhanced decomposition fostered by root exudates can actually explain the observed behavior. The complexity of the structural trends highlighted by the chemical characterization of DHS collected from planted and non-planted soils suggests that they should be further fractionated in order to isolate active fractions and allow a better characterization of their structure.

Besides confirming activation of genes involved in nitrate acquisition, this study demonstrated not only that the range of concentrations generally employed to investigate actions of HS on plants are indeed representative of agricultural field conditions but also the integrity of the use of the easily available WEHS in this type of studies.

## Data Availability Statement

All datasets generated for this study are included in the article/**Supplementary Material**.

## Author Contributions

All authors contributed to the study conception, design, data collection, analyses, and manuscript preparation. TV, LZ, SV performed the experiments. TV and LZ wrote the article, MN, SC, NT, RP supervised and completed the writing. TV, LZ, PC, SC, RP contributed to the study design. TV, LZ, SV, MC, NT, SC, MN contributed to the data analysis. All authors read and approved the final manuscript.

## Conflict of Interest

The authors declare that the research was conducted in the absence of any commercial or financial relationships that could be construed as a potential conflict of interest.

## Abbreviations

AC, active carbonates; BD, bulk density; CEC, cation exchange capacity; CF, conventional farming; C_org_, organic carbon; DHS, dissolved humic substances; DOC, dissolved organic carbon; DOM, dissolved organic matter; DON, dissolved organic nitrogen; FA, fulvic acids; FC, field capacity; HA, humic acids; N, nitrogen; NR, nitrate reductase; NiR, nitrite reductase; OF, organic farming; PWP, permanent wilting point; SMB, soil microbial biomass; SOM, soil organic matter; SWC, saturated water content; WEHS, water-extractable humic substances.

## Supplementary Material

Table S1 List of primers used for real-time RT-PCR analyses.
