# Supplementary material for: Biostimulant Action of Dissolved Humic Substances From a Conventionally and an Organically Managed Soil on Nitrate Acquisition in Maize Plants
Source: Front Plant Sci. 2020 Jan 15;10:1652. doi: 10.3389/fpls.2019.01652 (PMC6974922; doi:10.3389/fpls.2019.01652)
Supplement: Table S1 — List of primers used for real-time RT-PCR analyses. [file DataSheet_1.pdf]

# Supplementary Table 1

| Gene | *Accession Number* | *Primer sequences* | | *Efficiency* |
|---|---|---|---|---|
| | | *Forward (5'- -3')* | *Reverse (5'- -3')* | |
| ***ZmNRT2.1*** | AJ344451 | GATCGACGATCACCTATACCTC | GTGCTCCGTTGACATGAG | 74.7 % |
| ***ZmNRT2.2*** | AY659965.1 | GTGGTGTTCGTGCACTTCC | CTTCTCGTCGTCGTTCCACT | 83.6% |
| ***ZmMHA2*** | U09989 | TCCGACTGTTGTTTGTCGAG | CACCGACTCCATCCTCATCT | 87.1 % |
| ***ZmNADH:NR*** | M27821 | GGTCTTTGGAGGTGGAGGTGCTG | CTCTGGCTGCGTATTCAAACTCTCGT | 81.3 % |
| ***ZmNADPH:NR*** | X64446 | GGACCACGACATGCTCCTAA | GACCTTGCTGACCACGTACC | 89.4 % |
| ***ZmNiR*** | EU957616.1 | CTTCATGGGCTGCCTCAC | GTAGACGTCGGCCAGGTG | 91.5% |
| ***ZmRPL17*** | AF034948 | AAAGTCTCGCCACTCCAATG | ACGTCCAAGCCTTTCACATC | 80.0 % |
| ***ZmGADPH*** | XM_020551757 | CCTGCTTCTCATGGATGGTT | TGGTAGCAGGAAGGGAAGCA | 93.5 % |
| ***ZmTUA*** | NM_001174192 | AGGTCATCTCATCCCTGACG | TGAAGTGGATCCTCGGGTAG | 95.2 % |
